# Multiple colonization and dispersal events hide the early origin and induce a lack of genetic structure of the moss *Bryum argenteum* in Antarctica

**DOI:** 10.1002/ece3.6601

**Published:** 2020-08-05

**Authors:** Serena Zaccara, Jairo Patiño, Peter Convey, Isabella Vanetti, Nicoletta Cannone

**Affiliations:** ^1^ Department of Theoretical and Applied Sciences Insubria University Varese VA Italy; ^2^ Plant Conservation and Biogeography Departamento de Botánica Ecología y Fisiología Vegetal Universidad de La Laguna La Laguna Spain; ^3^ British Antarctic Survey Natural Environment Research Council High Cross Cambridge UK; ^4^ Department of Science and High Technology Insubria University Como CO Italy

**Keywords:** Antarctica, dispersal routes, Nunatak refugia, Palaeoclimate, phylogenetic patterns, transoceanic disjunctions

## Abstract

The dispersal routes of taxa with transoceanic disjunctions remain poorly understood, with the potential roles of Antarctica not yet demonstrated. Mosses are suitable organisms to test direct intra‐Antarctic dispersal, as major component of the extant Antarctic flora, with the cosmopolitan moss *Bryum argenteum* as ideal target species. We analyzed the genetic structure of *B. argenteum* to provide an evolutionary time frame for its radiation and shed light into its historical biogeography in the Antarctic region. We tested two alternative scenarios: (a) intra‐Antarctic panmixia and (b) intra‐Antarctic genetic differentiation. Furthermore, we tested for evidence of the existence of specific intra‐Antarctic dispersal routes. Sixty‐seven new samples (40 collected in Antarctica) were sequenced for ITS nrDNA and rps4 cpDNA regions, and phylogenetic trees of *B. argenteum* were constructed, with a focus on its Southern Hemisphere. Combining our new nrDNA dataset with previously published datasets, we estimated time‐calibrated phylogenies based on two different substitution rates (derived from angiosperms and bryophytes) along with ancestral area estimations. Minimum spanning network and pairwise genetic distances were also calculated. *B. argenteum* was potentially distributed across Africa and Antarctica soon after its origin. Its earliest intra‐Antarctic dispersal and diversification occurred during a warming period in the Pliocene. On the same timescale, a radiation took place involving a dispersal event from Antarctica to the sub‐Antarctic islands. A more recent event of dispersal and diversification within Antarctica occurred during a warm period in the Pleistocene, creating favorable conditions also for its colonization outside the Antarctic continent worldwide. We provide evidence supporting the hypothesis that contemporary populations of *B. argenteum* in Antarctica integrate a history of both multiple long‐range dispersal events and local persistence combined with in situ diversification. Our data support the hypothesis that *B. argenteum* has been characterized by strong connectivity within Antarctica, suggesting the existence of intra‐Antarctic dispersal routes.

## Graphical abstract

We analyze the genetic signature of the cosmopolitan moss *Bryum argenteum* in the Antarctic region and also at global scale. We reconstructed phylogenetic trees of *B. argenteum* at Antarctic scale applying molecular clock and ancestral areas reconstruction. This species was already occurring in Antarctica soon after its origin. Then, it colonized Antarctica several times during periods of climatic optima. The lack of genetic structure at intra‐Antarctic level could be due to local intra‐Antarctic genetic admixture favored by dispersal following colonization. Intra‐Antarctic dispersion may have occurred involving both the Transantarctic Mountains and the West Antarctic continental coastline

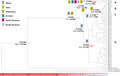

## 1 INTRODUCTION

Some plant species exhibit transoceanic disjunctions and, for species with disjunct distributions, their underlying dispersal routes and diversification patterns across space and time remain to be clarified. To explain the disjunct distributions of austral plants between South America and the western Pacific, Winkworth, Hennion, Prinzing, and Wagstaff (2015) hypothesized both out‐of‐Antarctica and intra‐Antarctic dispersal routes, identifying two potential periods warmer than today that could have supported such dispersal (Miocene, 23–5.33 Ma, and Pliocene, 5.33–2.58 Ma). However, while the potential for dispersal from the Antarctic continent is recognized, the identification of taxa that followed this pattern remains challenging due to the subsequent extinction of most of the Antarctic biota. Indeed, in most cases topologies based on, for instance, extant plant taxa are largely uninformative with respect to dispersal routes due to the fragmentation of the extant flora or to potentially incomplete sampling (Winkworth et al., 2015).

In the Antarctic region, the contemporary vegetation mainly comprises nonvascular cryptogams, with bryophytes and, in particular, mosses being the dominant terrestrial flora (Convey, 2017; Ochyra, Smith, & Bednarek‐Ochyra, 2008). Bryophytes are generally assumed to have considerable dispersal power (Patiño & Vanderpoorten, 2018), facilitated by the production of small sexual spores and asexual propagules, although sexual reproduction is observed frequently only at Antarctic locations where climatic conditions are less extreme, such as in Maritime Antarctica in which includes the Antarctic Peninsula and Scotia Arc archipelagos (Convey & Smith, 1993; Ochyra et al., 2008; Smith & Convey, 2002). Such life history traits promote widespread distribution and presence in extremely diverse terrestrial habitats throughout the world, from the tropics to the poles (Biersma et al., 2017; Huttunen, Kuznetsova, Li, Wang, & Ignatov, 2015; Lewis, Rozzi, & Goffinet, 2014; Patiño, Goffinet, Sim‐Sim, & Vanderpoorten, 2016).

Among bryophytes, *Bryum argenteum* Hedw. is a cosmopolitan moss, characterized by global distribution from tropical to polar latitudes, from sea‐level up more than 4,000 m altitude, and from pristine natural environments to heavily human‐impacted habitats (Bansal & Nath, 2013; Ingimundardottir, Weibull, & Cronberg, 2014; Kurschner, 1996; Puche & Gimeno, 2000). In Antarctica, *B. argenteum* has among the widest geographical distribution and highest abundance of the mosses present (Ochyra et al., 2008), thereby being an ideal model species to infer colonization routes and dispersal patterns. The historical biogeographic and recent demographic patterns of some Antarctic biota may have been facilitated by in situ persistence in refugial areas associated with geothermal activity, such as those located in the northern Antarctic Peninsula and Scotia Arc, Marie Byrd Land, and Victoria Land (Convey & Smith, 2006; Fraser, Terauds, Smellie, Convey, & Chown, 2014).

Recently, molecular phylogeographic studies have been applied to reconstruct the global distribution of *B. argenteum*, including its Antarctic range wherein the occurrence of multiple colonization events and in situ persistence have been proposed (Hills, Stevens, & Gemmill, 2010; Skotnicki, Mackenzie, Clements, & Selkirk, 2005), and higher genetic variation reported than in all other regions globally (Pisa et al., 2014). Building upon the multiple colonization event scenario proposed by Pisa et al. (2014) and using a newly enlarged dataset for Antarctica, in the current study we aim to clarify understanding of the historical biogeography of this species in the Antarctic continent and the surrounding regions.

To provide an evolutionary timeframe for the inter‐ and intra‐Antarctic dispersal and radiation events for *B. argenteum*, and to reconstruct its historical biogeography in this region, we generated time‐calibrated phylogenies based on two different models of substitution rates, inferred, respectively, from the Moss Tree of Life (Laenen et al., 2014) and from a broad meta‐analysis of molecular substitution rates in angiosperms (Kay, Whittall, & Hodges, 2006). More specifically, we assessed which of the following scenarios best explain the Antarctic population genetic structure and historical biogeography of *B. argenteum*:

Scenario I: There is no evidence of genetic structure, pointing to panmixia and/or strong connectivity across Antarctica;

Scenario II: There is strong genetic structure that matches the geographic pattern of Antarctic biogeographic regions, known as Antarctic Conservation Biogeographic Regions (ACBRs), for the Antarctic Peninsula (North‐West Antarctic Peninsula—NWAP; North‐East Antarctic Peninsula—NEAP; and Central‐South Antarctic Peninsula—CSAP) and for part of Continental Antarctica (Northern Victoria Land—NVLCA; Southern Victoria Land Continental Antarctica—SVLCA) (see Terauds et al., 2012; Terauds & Lee, 2016).

Furthermore, we hypothesized that patterns of genetic structuring would correspond to specific intra‐Antarctic dispersal routes, as suggested by Winkworth et al. (2015), in particular along the Transantarctic Mountains and/or the coast of continental Antarctica, with dispersal events occurring during periods of reduced ice cover (Cannone, Convey, & Guglielmin, 2013).

## 2 MATERIALS AND METHODS

### 2.1 Sampling strategy

**Figure 1:**
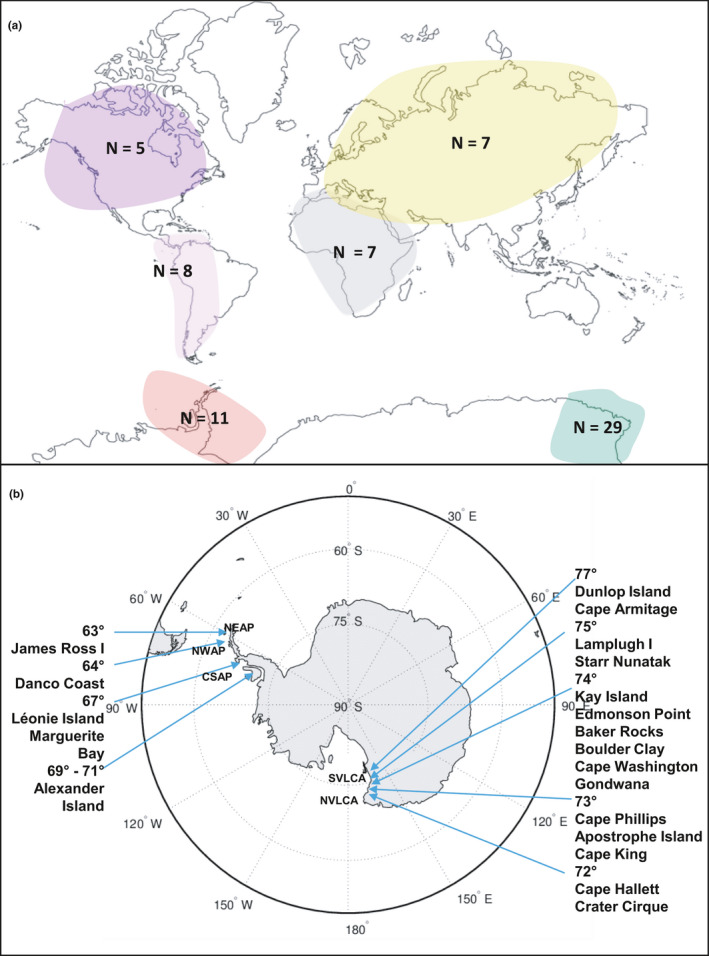
(a) Global map indicating the number of specimens of *B. argenteum* analyzed from each continent; Antarctic continent separated into Antarctic Peninsula (AP in red) and Continental Antarctica (CA in green). (b) Map of Antarctica, indicating locations and specific positions of each sample (i.e., latitude coordinates) as well as the Maritime Antarctic South Sandwich Islands (MASSI) and the relevant Antarctic Conservation Biogeographic Regions (ACBRs) (i.e., North‐West Antarctic Peninsula—NWAP; North‐East Antarctic Peninsula—NEAP, Central‐South Antarctic Peninsula—CSAP; Northern Victoria Land—NVLCA; and Southern Victoria Land—SVLCA, as detailed in Table 1)

Forty new Antarctic specimens of *B. argenteum* were obtained from Victoria Land, continental Antarctica (CA), and the Antarctic Peninsula (AP), during several field campaigns carried out between 1994 and 2003 by PNRA (Programma Nazionale Ricerche in Antartide) and BAS (British Antarctic Survey) researchers (see Table 1). Samples were collected along two latitudinal transects. Eleven samples were obtained from key geographic areas in the AP, including James Ross Island (63°S) and various locations at latitudes up to 72°S (Alexander Island). Twenty‐nine samples were collected in Victoria Land, providing data from 13 new sites between Cape Hallett (72°S) and Miers Valley (78°S) (Table 1, Figure 1b). To expand the study's coverage, seven further specimens of *B. argenteum* were provided by the New York Botanical Garden and 20 by the Missouri Botanical Garden, covering the Northern Hemisphere (five from North America and seven from Asia) and the Southern Hemisphere (seven from Africa and eight from South America; Figure 1a; Table 1).

**Table 1:** Original ITS and rps4 sequences provided in this study, for which collection features (i.e., Continent, sample ID, collection localities, geographic coordinates, and herbarium codes for BAS: British Antarctic Survey; INS: Insubria; MO: Missouri Botanical Garden; and NYBG: New York Botanical Garden) and molecular data (i.e., haplotype, bp length, and GenBank accession numbers) are indicated

Continent	Sample ID	Herbarium	Localities	Latitude–Longitude		ITS			rps4	
					hap	bp	GB code	hap	bp	GB code
Antarctic										
NEAP	JR2	INS	James Ross I	63°40′S 58°15′W	Hap1	691	MF152769	Hap4rps	624	MG651618
NEAP	JR3	INS	James Ross I	63°40′S 58°15′W	Hap9	693	MF152770			
NEAP	JR6	INS	James Ross I	63°40′S 58°15′W	Hap1	692	MF152771	Hap5rps	626	MG651619
NWAP	AAS877B	BAS	Danco Coast	64°08′S 64°08′W	Hap7	691	MF152774			
CSAP	AP89	BAS	Léonie Island	67°60′S 68°35′W	Hap6	767	KT947989	Hap6rps	627	KT964602
CSAP	AP46	BAS	Marguerite Bay	67°97′S 67°32′W	Hap1	1,025	KT947990	Hap6rps	624	KT964603
CSAP	AP468	BAS	Marguerite Bay	67°97′S 67°32′W	Hap4	1,042	KT947991	Hap6rps	626	MG651620
CSAP	AP20	BAS	Alexander Island	69°58′S 71°63′W	Hap1	758	KT947992			
CSAP	AP940	BAS	Alexander Island	71°88′S 68°25′W	Hap1	1,038	KT947993	Hap7rps	624	MG651621
CSAP	AP94	BAS	Alexander Island	71°88'S 68°25'W	Hap1	1,040	KT947994	Hap7rps	626	MG651622
CSAP	AAS12141	BAS	Alexander Island	71°73'S 72°68'W	Hap1	689	MF152772	Hap7rps	626	MG651623
NVLCA	VL25	INS	Cape Hallett	72°33'S 170°42'E	Hap1	758	KT947995	Hap7rps	624	MG651624
NVLCA	VL34	INS	Crater Cirque	72°38'S 169°22'E	Hap1	1,040	KT947996	Hap7rps	624	MG651625
NVLCA	VL242	INS	Cape Phillips	73°07'S 169°60'E	Hap1	1,033	KT947997	Hap7rps	624	KT964604
NVLCA	VL531	INS	Apostrophe I	73°31′S, 167°26′E	Hap10	679	MG651608	Hap8rps	626	MG651626
NVLCA	VL7	INS	Apostrophe I	73°31′S, 167°26′E	Hap10	698	MF152778	Hap8rps	621	MG651627
NVLCA	VL8	INS	Apostrophe I	73°31′S, 167°26′E	Hap1	690	MF152779	Hap7rps	624	MG651628
NVLCA	VL152	INS	Cape King	73°35′S 166°37′E	Hap1	671	MG651609	Hap7rps	624	MG651629
NVLCA	VL15	INS	Cape King	73°35′S 166°37′E	Hap1	1,035	KT947998	Hap7rps	624	KT964606
NVLCA	VL10	INS	Cape King	73°58′S 166°62′E	Hap1	751	KT947999	Hap7rps	584	KT964607
NVLCA	VL95	BAS	Kay Island	74°07′S 165°32′E	Hap1	771	KT948000	Hap7rps	624	KT964608
NVLCA	VL99	BAS	Kay Island	74°07′S 165°32′E	Hap1	757	KT948001			
NVLCA	VL07	INS	Edmonson Point	74°19′S 165°07′E	Hap11	1,039	KT948002	Hap8rps	615	KT964609
NVLCA	VL26	INS	Edmonson Point	74°19′S 165°07′E	Hap1	752	KT948003	Hap9rps	610	KT964610
NVLCA	VL1	INS	Edmonson Point	74°19′S 165°07′E	Hap10	698	MF152775	Hap8rps	624	MG651630
NVLCA	VL2	INS	Edmonson Point	74°19′S 165°07′E	Hap10	698	MF152780	Hap8rps	623	MG651631
NVLCA	VL4	INS	Edmonson Point	74°19′S 165°07′E	Hap1	690	MF152777	Hap10rps	621	MG651632
NVLCA	VL5	INS	Edmonson Point	74°19′S 165°07′E	Hap10	698	MF152776	Hap8rps	621	MG651633
NVLCA	VL96	INS	Baker Rocks	74°23′S 164°75'E	Hap1	671	MG651610	Hap7rps	624	KT964611
NVLCA	VL953	INS	Baker Rocks	74°23′S 164°75′E	Hap1	857	KT948004	Hap7rps	624	KT964612
NVLCA	VL37	INS	Boulder Clay	74°44′S 164°01′E	Hap10	921	KT948005	Hap8rps	627	KT964613
NVLCA	VL43	INS	Boulder Clay	74°44′S 162°01′E	Hap1	758	KT948006	Hap7rps	612	KT964614
NVLCA	VL98	INS	Cape Washington	74°48′S 162°42′E	Hap1	766	KT948007	Hap7rps	624	KT964615
NVLCA	VL97	INS	Gondwana	74°68′S 164°10′E	Hap5	761	KT948008	Hap7rps	625	KT964616
NVLCA	VL974	INS	Gondwana	74°68′S 164°10′E	Hap1	671	MG651611	Hap7rps	559	MG651634
SVLCA	VL24	INS	Lamplugh I	75°33′S 162°57′E	Hap68	603	MG651612	Hap8rps	625	MG651635
SVLCA	00256B	BAS	Edmonson Point	74°33′S 165°13′E	Hap1	673	MK234244	Hap7rps	559	MK234294
SVLCA	VL51	INS	Starr Nunatak	75°53′S 162°35′E	Hap1	867	KT948009	Hap7rps	624	MG651636
SVLCA	VL29	INS	Dunlop Island	77°14′S 162°28′E	Hap1	1,034	KT948009	Hap7rps	559	MG651637
SVLCA	AAS4016	BAS	Cape Armitage	77°85′S 166°67′E	Hap1	690	MF152773	Hap7rps	625	MG651638
*Asia*	35669	MO	China, Yunnan	27°21′N 100°09 E	Hap85	6,778	MK234234	Hap11rps	559	MK234284
	34878	MO	China, Sichuan	30°36′N 102°43′E	Hap87	683	MK234235	Hap13rps	559	MK234285
	951081	MO	China, Xinjiang	43°07′N 84°45′E	Hap56	679	MK234248			
	508A	MO	Kazakhstan	NA NA	Hap36	677	MK234236	Hap26rps	559	MK234286
	10422	MO	Kazakhstan	43°00′N 77°00′E	Hap89	519	MK234237	Hap13rps	559	MK234287
	1229466	NYBG	Turkey	NA NA	Hap29	680	MG651616	Hap13rps	621	MG651644
	1048324	NYBG	Buthan, Tongsa	27°29′N 90°30′E	Hap45	510	MG651617	Hap13rps	621	MG651645
*Africa*	30591	MO	Guinea	07°40′N 8°23′W	Hap94	673	MK234238	Hap24rps	559	MK234288
	12503	MO	Malawi		Hap10	688	MK234239	Hap6rps	559	MK234289
	12555	MO	Malawi		Hap36	677	MK234240	Hap12rps	559	MK234290
	703	MO	Namibia		Hap36	677	MK234245	Hap12rps	559	MK234295
	3589	MO	South Africa	28°34′S 23°49′E	Hap97	666	MK234241	Hap17rps	559	MK234291
	6963A	MO	South Africa		Hap26	663	MK234242	Hap17rps	559	MK234292
	144	MO	Tanzania	04°15′S 37°59′E	Hap45	687	MK234243	Hap13rps	559	MK234293
*North America*	69305	NYBG	Alaska	68°30′N 149°25′W	Hap56	679	MG651613	Hap11rps	621	MG651639
	69308	NYBG	Alaska	55°18′N 160°22′W	Hap56	549	MG651614	Hap11rps	624	MG651640
	69321	NYBG	Canada, Nunavut	80°03′N 88°75′W	Hap63	504	KF952889*	Hap11rps	621	MG651641
	69322	NYBG	Canada, Nunavut	79°48′N 85°26′W	Hap56	504	KF952878*	Hap11rps	622	MG651642
	69341	NYBG	Canada, Nunavut	NA NA	Hap29	680	MG651615	Hap12rps	625	MG651643
*South America*	9148	MO	Peru	13°00′S 72°31′W	Hap87	684	MK234228	Hap13rps	559	MK234278
	572	MO	Bolivia	16°24′S 67°53′W	Hap72	662	MK234229	Hap12rps	559	MK234279
	2143	MO	Bolivia	19°05′S 65°04 W	Hap35	681	MK234230	Hap13rps	559	MK234280
	2620	MO	Bolivia	20°13′S 64°27′W	Hap75	499	MK234231	Hap16rps	559	MK234281
	4154	MO	Bolivia	21°33′S 67°36′W	Hap55	677	MK234232	Hap18rps	559	MK234282
	30425	MO	Chile	37°00′S 72°59′W	Hap12	521	MK234246			
	34678	MO	Chile	36°39′S 71°16′W	Hap16	675	MK234233	Hap12rps	559	MK234283
	16953	MO	Chile	54°07′S 68°40′W	Hap12	680	MK234247			

Notes

Antarctic Conservation Biogeographic Regions (ACBRs) are also detailed: North‐West Antarctic Peninsula (NWAP); North‐East Antarctic Peninsula (NEAP), Central‐South Antarctic Peninsula (CSAP); Northern Victoria Land Continent Antarctica (NVLCA); and Southern Victoria Land Continent Antarctica (SVLCA).

### 2.2 Molecular data

We analyzed the internal transcribed spacer (nrITS) region and the chloroplast gene rps4 (cpDNA) to obtain data comparable with the available literature. From each collected gametophyte, total genomic DNA was extracted using the PowerPlant DNA Isolation Kit (Qiagen, Inc.) and Proteinase K digestion. PCR amplification of the ITS region was carried out using primers specifically redesigned for *B. argenteum* (Pisa et al., 2014), while rps4 was amplified using the primers RPS5 (Nadot, Bittar, Carter, Lacroix, & Lejeune, 1995) and TRNAS (Buck, Goffinet, & Shaw, 2000). Each PCR reaction contained 10 μL reaction volume with 0.2 μM concentration of each primer, 5 μl QIAGEN Multiplex (QIAGEN multiplex PCR kit), and 2 μl extracted DNA. Both amplifications were performed using a thermocycler (BIO RAD – T100 Thermal Cycler) with the following protocol: 15 min at 95°C followed by 40 cycles each of 94°C for 30 s, 56°C for 90 s and 72°C for 90 s, and finally 10 min at 72°C.

PCR products were purified with EuroSap enzymes (Euroclone) and directly sequenced in both forward and reverse directions (MACROGEN Inc. Amsterdam, The Netherlands; http://www.macrogen.org) using a 3730XL DNA Sequencer. The sequences were aligned using Bioedit ver.7.0.9 (Hall, 1999) and edited by eye allowing gaps where necessary to conserve homology among sequences. All the sequences generated in this study, deposited in GenBank under the accession numbers KT947989–KT948009, MF152769–MF152780, MK234228–MK234295 and MG651608–MG651645 (Table 1), were grouped into haplotypes using DnaSP ver. 5.10 (Librado & Rozas, 2009). The nrITS region has been more extensively analyzed than the cpDNA region in *B. argenteum*, and we therefore focused on this specific nuclear marker for the biogeographical analyses since it provides a more comprehensive biogeographical context. The nrITS sequences obtained in the present study (67) were aligned with all globally available data for the species, which gave a total of 149 nrITS sequences (Hills et al., 2010; Pisa et al., 2014; Rankin et al., 2017; Skotnicki et al., 2005; Table S1). Hereafter, we refer to this alignment as the global nrITS dataset.

### 2.3 Phylogenetic analyses

The phylogeny of *B. argenteum* was independently reconstructed using the nrITS and cpDNA datasets obtained in the present study (Table 1). The phylogenetic trees were rooted using closely related congeneric species as outgroups (*B. arcticum *Bruch & W. P. Schimper, *B. funckii *Schwägr.*, B. recurvulum *Mitten, *B. paradoxum *Schwaegrichen, and *B. apiculatum *Schwaegrichen). The selection of these outgroup species was based on recent phylogenetic work on the genus *Bryum* s.l. (Hills et al., 2010; Pisa et al., 2014; Skotnicki et al., 2005). Maximum likelihood (ML) analyses were performed using the GTR + G and HKY + I replacement models of nucleotide evolution for nrITS and cpDNA, respectively, as recommended by jModelTest software (Posada, 2008) selected under the Akaike information criterion. Confidence values of the nodes were estimated using 1,000 bootstrap replicates. The analyses were computed using PAUP 4.0b (Swofford, 2002) and GARLI ver. 1.0 (Bazinet, Zwickl, & Cummings, 2014; Zwickl, 2006).

### 2.4 Timeframe and ancestral area estimation

To provide an evolutionary timeframe for the historical biogeography of *B. argenteum* in Antarctica, a time‐calibrated phylogeny was estimated. The strict and the uncorrelated relaxed molecular clocks (lognormal) were tested using BEAST ver. 1.8.4 (Drummond, Suchard, Xie, & Rambaut, 2012). Because the inclusion of identical sequences results in many zero length branches at the tip of the tree and can cause the model to over‐partition the dataset (Reid & Carstens, 2012), we performed the BEAST analyses at the level of haplotypes. In the absence of fossil records of *B. argenteum*, we applied two calibration approaches based on two distinct nucleotide substitution rates assessed for (calibration I) herbaceous angiosperms (mean = 4.13 × 10^–3^, *SD* = 4 × 10^–4^ substitutions/site/million years (Kay et al., 2006; for a review see Villarreal and Renner (2014)), as previously used by Pisa et al. (2014), and (calibration II) mosses (mean 4.45 × 10^–4^, *SD* 1.77 × 10^–6^) as inferred from the Moss Tree of Life (Laenen et al., 2014), with a modification in order to broaden the parameter *SD* (1.77 × 10^–3^) and allow testing whether the priors of the two calibration approaches (I and II) could provide posterior distributions (i.e., 95% highest posterior density interval) for the estimated clade ages that were congruent (i.e., overlap). For each analysis, Bayesian reconstructions were conducted using four different tree priors, including Yule and birth–death process priors, as well as coalescent priors such as the models Constant Size and Bayesian Skyline. For each analysis, we conducted two independent runs to ensure convergence of the MCMC. We then compared their posterior distributions using the marginal likelihood estimate (MLE) of each model, estimated from stepping‐stone sampling (SS) and path sampling (PS). We performed MLE using SS with 150 path steps, each with a chain length of one million iterations, and the other parameters were set by default. We directly calculated the log‐Bayes factors from MLEs and used BF to compare the support of all the models tested. We considered BF values above 2 to indicate that one model was significantly favored over another. In both cases (calibrations I and II), results from PS and SS MLEs were very similar, and a constant size tree model under an uncorrelated relaxed molecular clock model was selected (see Table S2).

To provide evidence of inter‐ and intra‐Antarctic biogeographic range evolution, we used the R package BioGeoBEARS (BioGeography with Bayesian Evolutionary Analysis in R Scripts) (Matzke, 2014) to perform ancestral area estimations based on the two time‐calibrated MCC trees obtained from BEAST (calibrations I and II) (see Table S3). After removing the outgroups, we performed the ancestral area estimations across the ingroup. We applied three different biogeographical models (DEC, DIVALIKE, and BAYAREA) under a maximum likelihood framework and compared how well they fitted the data using the Akaike information criterion corrected for small sample size (AICc).

### 2.5 Molecular indices and genetic structure of samples from Antarctic regions

The population genetic structure of *B. argenteum* was assessed by specifically using a restricted multiple alignment of 40 new sequences collected in Antarctic regions (from 63° to 77°S) plus a further 29 sequences available from the literature which also included the sub‐Antarctic islands (Hills et al., 2010; Pisa et al., 2014; Rankin et al., 2017; Skotnicki et al., 2005), giving an overall latitudinal range from 46° S to 78° S (Table S1). This multiple alignment covered the AP (*n* = 15), CA (*n* = 48), four sequences from sub‐Antarctic islands (SAI) at 46° S (Prince Edward Island, Crozet Islands and Possession Island) and two from the northern Maritime Antarctic South Sandwich Islands (MASSI) at 57° S (Candlemas Island) (see Table S1).

For visual inspection, a minimum spanning network (MSN) was created using statistical parsimony criteria as implemented in TCS ver. 1.18 (Clement, Posada, & Crandall, 2000). Descriptive statistics of haplotype and nucleotide diversity were obtained using DnaSP and Arlequin ver. 3.5.1.3 (Excoffier & Lischer, 2010). Estimated values for the entire Antarctic dataset, and for AP and CA considered separately, included the number of sequences (N), number of haplotypes (n), nucleotide diversity (π), and haplotype diversity (H). Levels of pairwise genetic differentiation between the Antarctic regions (i.e., CA and AP) and between local biogeographic classification (i.e., NEAP, NWAP, and CSAP for AP; and NVLCA and SVLCA for CA) were estimated as pairwise genetic distances (Φ_ST_), and their significance tested by 1,000 random permutations.

## 3 RESULTS

### 3.1 *Phylogeny of* Bryum argenteum

The complete alignment obtained from the 67 new nrITS sequences consisted, excluding outgroups, of a total length of 524 bp, with 55 indels and 29 variable positions of which 15 were parsimony informative. For the cpDNA alignment, we obtained 60 sequences, with the complete alignment consisting of 558 bp, including 26 variable sites of which 14 were parsimony informative.

Among the 67 nrITS sequences, 34 haplotypes were identified and nine of these were found in Antarctic regions (Table 1). *Hap1* was the most frequent (*n* = 27) and widespread haplotype in Antarctic regions, found from 63°S (in AP) to 77°S (CA). The other eight Antarctic nrITS haplotypes were found exclusively in either AP (*Hap4, Hap6*, *Hap7*, and *Hap9*) from locations between 63 and 67°S, or CA (*Hap5, Hap10*, *Hap11*, and *Hap68*) between 73 and 75°S). Thirteen haplotypes were identified among the cpDNA sequences, seven of which occurred in Antarctic regions. The most frequent cpDNA haplotype (*Hap7rps*, *n* = 21) was again present in both AP and CA (*n* = 3 and *n* = 18, respectively), while the other six haplotypes were recorded from a single region: *Hap8rps*, *Hap9rps,* and *Hap10rps* in CA and *Hap4rps*, *Hap5rps,* and *Hap6rps* in AP (see Table 1).

**Figure 2:**
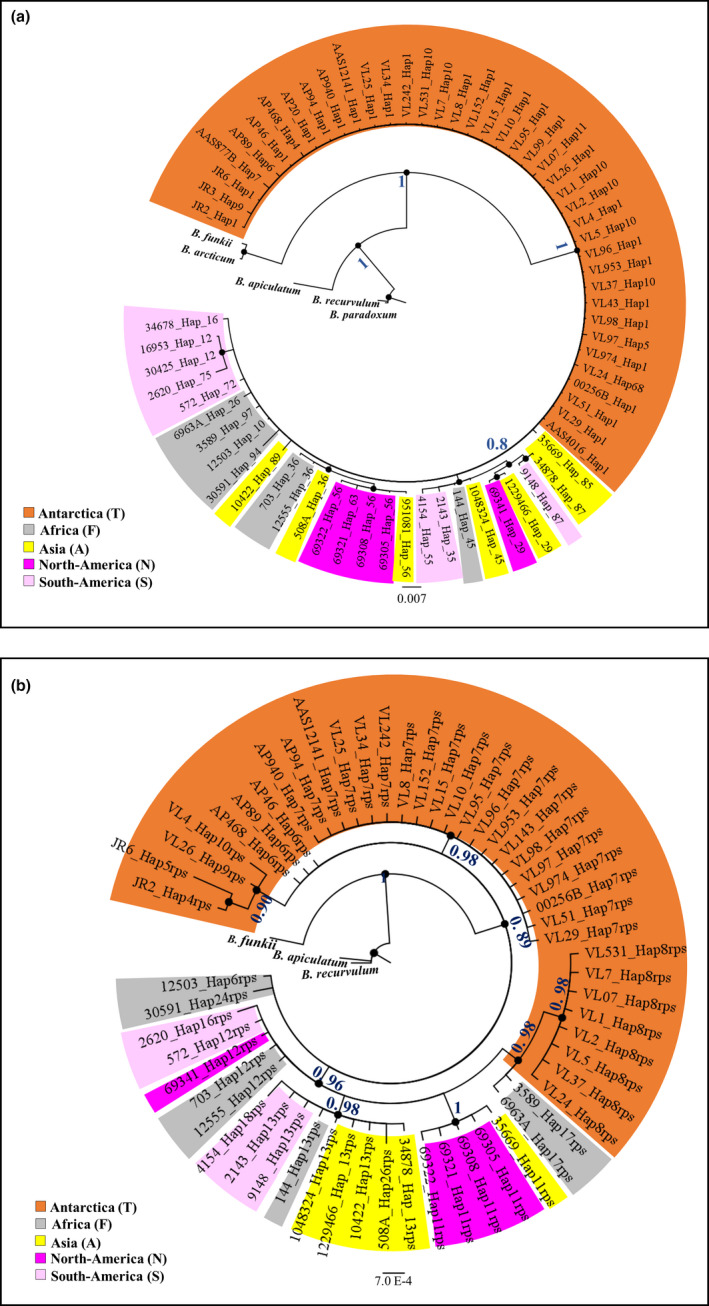
Phylogenetic trees based on (a) ITS nrDNA and (b) rps4 cpDNA sequences, as detailed in Table 1. Statistical support is expressed both as posterior probability and bootstrap values (>0.8 and 80, respectively). The trees were rooted on *B. recurvulum*, *B. apiculatum,* and *B. funkii* for both markers, and also on *B. articum* and *B. paradoxum* for the ITS dataset only. The geographic origins are as follows: Antarctica (T), Africa (F), Asia (A), North America (N), South America (S), and Europe (E)

In both phylogenetic trees (based on nrITS and cpDNA sequences), the Antarctic samples were not clustered in monophyletic clades (Figure 2a, b). The nrITS tree topology showed polytomy between Antarctic sequences (Figure 2a), while the cpDNA tree had a more complex topology in which Antarctic samples were distributed in three clades (pp> 0.9) (Figure 2b) (See Table 1).

**Figure 3:**
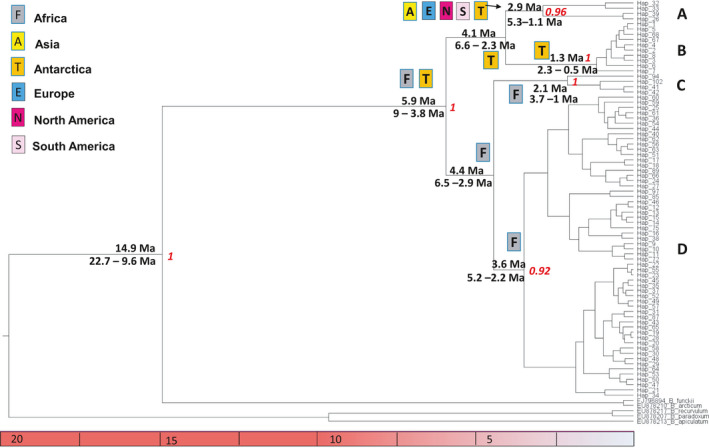
BEAST chronogram of *B. argenteum* ITS nrDNA haplotypes based on bryophyte substitution rate (calibration II) under the best fitting model (see Table S2). The geographic distribution of haplotypes is detailed in Table 1 and Table S1. Results of ancestral areas analysis based on DIVALIKE biogeographical model (see Table S3) in relation to the BEAST chronogram are also indicated. Posterior probability values at nodes (pp> 0.90) are indicated. The 95% highest posterior density (HPD) intervals are provided in Table 2. The geographic origins are as follows: Antarctica (T), Africa (F), Asia (A), North America (N), South America (S), and Europe (E)

The nrITS phylogeny tree of Antarctic samples (Figure 2a) was resolved analyzing the global nrITS dataset (see Table S1). In particular, the global nrITS phylogenetic tree identified four statistically supported clades: clade A (pp = 0.97), clade B (pp = 1), clade C (pp = 1), and clade D (pp = 0.91) (Figure 3), with three of them (A, B, and D) including Antarctic haplotypes. Clade A included a number of widely distributed haplotypes, present in Europe, North America, Asia, and Antarctica, with the latter only identified from the Danco Coast (*Hap26*; Pisa et al., 2014) located in the AP (Figure 3; Table S1). Most members of Clade B were new haplotypes present only in CA and AP (*Hap2‐8, Hap68*) with the exception of *Hap1*, the most common and widespread haplotype, that was recorded in Antarctica and also in Asia, Europe, and North America. Clade C included haplotypes occurring in Asia, South America, and Africa. Clade D included haplotypes detected in all continents, including new Antarctic haplotypes from both AP (*Hap9*) and CA (*Hap10*, *Hap11*, and *Hap64*) regions as well from the SAI (*Hap13*, *Hap16*, *Hap17*, and *Hap21*).

### 3.2 Timeframe and ancestral area estimation

The phylogenetic reconstructions based on the two different schemes of substitution rates obtained from angiosperms (calibration I in Fig. S1) and mosses (calibration II in Figure 3) provided a congruent timeframe (see Table 2), dating the species’ origin at 15.4 Ma (95% HPD: 21.0–10.7 Ma; pp = 1) or 14.9 Ma (95% HPD: 22.6–9.6 Ma; pp = 1), respectively. The first lineage split within the species was inferred to have occurred around 5.7 Ma (95% HPD: 7.7–4.0 Ma; pp = 1) or 5.9 Ma (95% HPD 9.0–3.8 Ma; pp = 1), respectively. The ancestral area analyses using the two BEAST chronograms selected DIVALIKE as the best‐fit model (Table S3). Both analyses indicated that the most recent common ancestor of *B. argenteum* was potentially distributed across Africa and Antarctica, although the calibration I analysis also indicated its presence in Asia and North America (See Table 2, Figure 3; Fig. S1). Both dating approaches generated closely similar timeframes for the diversification of each of the four clades, although the more recent internal splits (A vs. B and C vs. D) were not significantly supported (pp < 0.8). Following the calibration I and calibration II schemes, the clade divergence events were dated as follows (Table 2, Fig. S1, Figure 3): (a) clade A, 2.9 Ma in both schemes (95% HPD: 5.3–1.1 Ma or 4.7–1.3 Ma, respectively, both pp> 0.90); (b) clade B, 1.3 Ma (95% HPD: 2.3–0.5; pp> 0.91) or 1.2 Ma (95% HPD: 2.1–0.5 Ma; pp = 1), respectively; (c) clade C, 2.1 Ma in both schemes (95% HPD: 3.7–1.0 or 3.4–1.0 Ma, respectively, both pp> 0.90); and (d) clade D, 3.6 Ma (95% HPD: 5.2–2.2; pp> 0.90) or 3.4 Ma (95% HPD: 4.9–2.3; pp> 0.90), respectively.

**Table 2:** Timeframes provided by molecular clock analyses based on: i) angiosperm substitution rate (calibration I) and ii) bryophyte substitution rate (calibration II)

	Angiosperm substitution rate			Bryophyte substitution rate		
	Mean age in Ma	95% HPD	Ancestral areas	Mean age in Ma	95% HPD	Ancestral areas
**Origin of *Bryum argenteum***	**15.4**	**21–10.7**		**14.9**	**22.7–9.6**	
**Split A/B–C/D**				**5.9**	**9.0–3.8**	**F; T**
**Split A–B/C/D**	**5.7**	**7.7–4**	**F; A; N; T**			
Split C/D				4.4	6.5–2.9	F
Split B/C–D	4.9	6.6**–**3.5	F			
Split A/B				4.1	6.6**–**2.3	T
Split B/C	4.0	5.7**–**2.5	F			
**Clade A** ^a^	**2.9**	**4.7–1.3**	**A**	**2.9**	**5.3–1.1**	**A; E; N; S; T**
**Clade B** ^a^	**1.2**	**2.1–0.5**	**T**	**1.3**	**2.3–0.5**	**T**
**Clade C** ^a^	**2.1**	**3.4–1.0**	**F**	**2.1**	**3.7–1.0**	**F**
**Clade D** ^a^	**3.4**	**4.9–2.3**	**F; N**	**3.6**	**5.2–2.2**	**F**

Notes

For each of the analyses, the mean divergences for selected nodes are given in millions of years (Ma) together with their 95% HPD. Ancestral areas in relation to the BEAST chronogram are also reported. The geographic areas (see Figure 3) are as follows: Antarctica (T), Africa (F), Asia (A), North America (N), South America (S), and Europe (E). In bold: internal node with pp > 0.9.

^a^ Complete overlap of clade structure.

Ancestral area estimates based on both BEAST chronograms identified the same ancestral origins for clade B (Antarctica) and clade C (Africa). The two analyses, however, identified different origins for clades A and D. In the calibration I chronogram, clade A originated from Asia, while in the calibration II chronogram, it originated from multiple continents including, but not restricted to, Asia (Table 2, Figure 3; Figure S1). Clade D showed an African origin in both chronograms, but a North American origin was also inferred in the calibration I chronogram (Table 2, Figure 3; Figure S1).

### 3.3 Intra‐Antarctic genetic diversity

**Figure 4:**
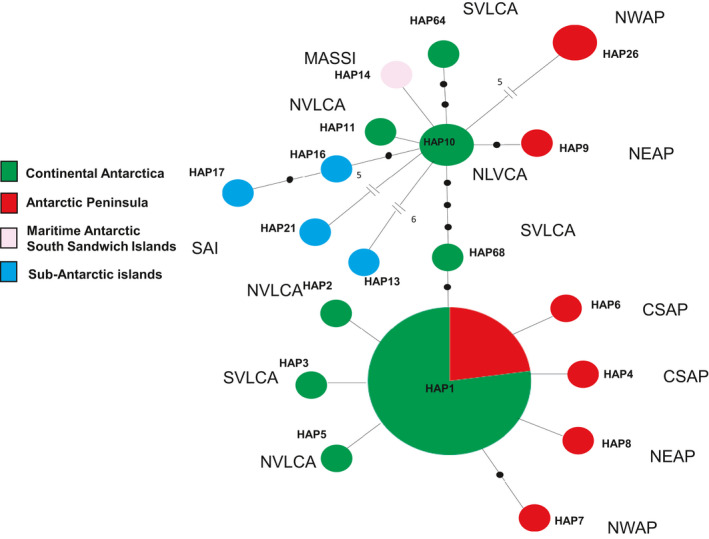
Minimum spanning network of ITS dataset from the Antarctic continent, indicating Antarctic Peninsula (AP in red), Continental Antarctic (CA in green), Maritime Antarctic South Sandwich Islands (MASSI in pink), and sub‐Antarctic islands (SAI in blue). Antarctic Conservation Biogeographic Regions (ACBRs) are also detailed: North‐West Antarctic Peninsula (NWAP); North‐East Antarctic Peninsula (NEAP); Central‐South Antarctic Peninsula (CSAP); Northern Victoria Land (NVLCA); and Southern Victoria Land (SVLCA). The black dots represent mutational steps. The detailed geographic positions are listed in Table 1 and Table S1

The species’ phylogeographic structure within Antarctica is illustrated in Figure 4. A star‐like structure was clear around the central position of *Hap1*, present and widespread in AP and CA, from which six haplotypes radiated with a single mutational step and one haplotype (*Hap7*) with two mutational steps. A second haplotype (*Hap10*) was at the center of a similar pattern, from which radiated *Hap11* and *Hap64* (located in CA) and *Hap9* and *Hap26* (located in AP). Both South Sandwich Islands (MASSI, Candlemas I.) and Sub‐Antarctic islands (SAI; Prince Edward I., Crozet I. and Possession I.) radiated from *Hap10* with between one (*Hap14*) and seven (*Hap 13*) mutational steps (Figure 4). There were six mutational steps between *Hap10* and *Hap1*, giving the overall network a dumbell shape (Figure 4).

Haplotype (H) and nucleotide (π) diversities in Antarctica were 0.54 and 0.025, respectively, while the H and π values in AP and CA ranged between 0.46 and 0.73 and between 0.023 and 0.033, respectively (Table 3). Pairwise Ф_ST_ comparisons between the two Antarctic regions were not significant.

**Table 3:** Molecular indices calculated for the Antarctic continent and its main regions (AP and CA), as detailed in Table 1

Molecular indices	Antarctic	AP 64°S–71°S	CA 72°S–78°S
*N* (*n*)	63 (14)	15 (7)	48 (8)
H ± St Dev	0.54 ± 0.07	0.73 ± 0.12	0.46 ± 0.08
π ± St Dev	0.025 ± 0.013	0.033 ± 0.02	0.023 ± 0.011

Notes

*N* = sample size, *n* = number of haplotypes

Analyzing genetic differentiation separately within CA and AP, significant Ф_ST_ values (*p* < .05) were only recorded in the North‐West Antarctic Peninsula (NWAP) (see Table 3). This area was significantly differentiated from both regions within Victoria Land (Ф_ST_ = 0.329, *p* < .05 and Ф_ST_ = 0.636, *p* < .01, from NVLCA and SVLCA, respectively), and from the Central South Antarctic Peninsula (CSAP; Ф_ST_ = 0.655, *p* < .01; Table 4).

**Table 4:** Pairwise Ф_ST_ comparisons among the ACBRs recognized in the Antarctic Peninsula (AP) and in Continental Antarctica (CA) and their significance after 1,000 permutations, indicated as follows: * *p* < .05; ** *p* < .01. Geographic regions are as given in Table 1

Antarctic zone	ACBRs	NEAP	NWAP	CSAP	NVLCA	SVLCA
AP	NE	–				
	NW	*0.305*	–			
	CS	*0.184*	0.655**	–		
CA	NVL	*0.001*	0.329*	*0.101*	*–*	
	SVL	*0.092*	0.636**	*0.043*	*0.085*	–

Note

In italics: no significant difference.

## 4 DISCUSSION

### 4.1 Phylogeny of B. argenteum

The phylogenetic tree and biogeographic evidence, achieved by comparing dating analyses using two different calibration schemes based on angiosperm and moss substitution rates, in combination with ancestral area estimations, show genetic signatures supporting the hypothesis that *B. argenteum* was present in both Antarctica and Africa (with calibration I also indicating presence in Asia and North America) soon after the species’ origin. However, the estimates of ancestral areas were equivocal toward the deepest nodes, not allowing robust conclusions to be drawn about the origin of the species, as previously found by Pisa et al. (2014). The current study outcomes, based on an enlarged Antarctic dataset in comparison with previous studies, suggest that *B. argenteum* was present in Antarctica soon after the species’ origin, most likely in the mid‐Miocene, and before the differentiation of its major clades. We then infer that the earliest intra‐Antarctic dispersal and diversification events (clade D) may have occurred during the mid‐Pliocene (around 3.5 Ma), likely coinciding with the extended Pliocene warming period (between 5.3 and 3.6 Ma; Salzmann et al., 2011). The molecular biogeographic footprint of clade D suggests that Antarctic mosses may have experienced geographic and genetic isolation, plausibly linked to cooling events that occurred in the late Pliocene and early to mid‐Pleistocene (Head & Gibbard, 2015) that likely erected climatic barriers. From the ancestral haplotype of clade D (*Hap10*), we identified several “private” haplotypes that appear to have radiated in situ in both the AP and CA. Members of this clade are also present on the SAI, sharing *Hap10* as a common ancestor. This supports a hypothesis of colonization from the AP or CA, and is consistent with evidence from *Sanionia uncinata* (Hedenäs, 2012) and the bipolar moss species *Polytrichum juniperinum* (Biersma et al., 2017). We recognize it is possible that some Antarctic biota could have survived on the sub‐Antarctic islands (particularly South Georgia) or volcanic South Sandwich Islands during glacial periods, from where they may have then recolonized continental Antarctica during warmer interglacials (Rogers, 2007); however, there is very little evidence available supporting this for terrestrial biota, other than for the South Sandwich Islands (Convey, Biersma, Casanova‐Katny, & Maturana, 2020; Convey & Smith, 2006).

The major Pliocene warming event between 5.3 and 3.6 Ma (Salzmann et al., 2011) may also have promoted the differentiation of clade A, although this has only very limited presence in Antarctica, and is currently only identified from a heavily glaciated area of the north‐western AP (Danco Coast, *Hap26;* Pisa et al., 2014). Our findings point to a recent history of dispersal and diversification events within Antarctica for clade B, which is reflected by the widespread presence in Antarctica of haplotypes restricted to this clade (other than the widespread *Hap1*). This second intra‐Antarctic evolutionary differentiation originated from two basal haplotypes (*Hap6* and *Hap7*) recorded exclusively from two locations on the AP (Danco Coast and Léonie Island), emphasizing that these geographic areas of the north‐western and central‐south Antarctic Peninsula played an important role in past Antarctic colonization events. These locations may be indicators of the regional presence of glacial refugia, in particular as consistent with the presence of areas of known high floristic richness (e.g., Léonie and Lagotellerie Islands within Marguerite Bay; Cannone, Convey, & Malfasi, 2018; the Berthelet Islands, Cape Primavera, Cape Tuxen, in the general region of the Danco Coast).

Clade B was inferred to have diverged around 1.2–1.3 Ma (see Table 2), probably exploiting favorable conditions that occurred during multiple short interglacial periods in the Pleistocene (for instance one estimated at 1.07 Ma; Swanger, Marchant, Schaefer, Winckler, & Head, 2011). *Hap1* may have originated during this specific interglacial, with wide dispersal also being favored by partial collapse of Antarctic ice sheets (Pollard & DeConto, 2009; Swanger et al., 2011), and favorable conditions for the species’ dispersal and colonization outside the Antarctic continent worldwide (see Table S1). Subsequent periods of cooling and glacial maxima may again have resulted in isolation, promoting in situ radiation from the widespread *Hap1*, consistent with the star‐like Antarctic haplotype network of this clade.

The complex history of Antarctic colonization here recorded in *B. argenteum* and its persistence through glacial periods within the continent complements recent observations in multiple groups of terrestrial biota (Biersma et al., 2017; Convey et al., 2008, 2018, 2020; McGaughran, Tearuds, Convey, & Fraser, 2019), including many invertebrate groups, lichens, and other mosses that currently occur in habitats and areas that are also commonly characterized by *B. argenteum*. These important components of the Antarctic biota must therefore have had environmentally suitable refugia available to them (Convey et al., 2018, 2020). However, the precise locations of such refugia remain unknown (Pugh & Convey, 2008), as does the degree of connectivity or isolation between individual locations.

### 4.2 Intra‐Antarctic genetic structure and dispersal routes

In a harsh climatic context such as that of Antarctica, genetic bottlenecks might be expected as a result of colonization events followed by geographical isolation and survival without demographic expansion, likely leading to biogeographic differentiation at an intra‐Antarctic scale. However, although limited by relatively small sample sizes and intra‐Antarctic coverage, our data indicate limited genetic differentiation between the AP and CA, providing some evidence for panmixia within Antarctica for this moss species and at least partially supporting our initial Scenario I.

Our analyses also provide support for the existence of intra‐Antarctic dispersal routes between the Antarctic Peninsula and Continental Antarctica, which could contribute to the overall lack of Antarctic population genetic structure in *B. argenteum*. Lack of evidence supporting intra‐Antarctic genetic differentiation could be related to the occurrence of intra‐Antarctic dispersal events following colonization, admixing the genetic differentiation signal provided by older and region‐specific colonization events. Although some basal haplotypes are restricted exclusively to the AP region, the widespread distribution of *Hap1* and the lack of genetic structure among the ACBRs support the existence of connection by dispersal routes between Victoria Land (CA) and the AP (Cannone et al., 2013; Ochyra et al., 2008). Other moss species such as *Ceratodon purpureus* (Hedw.) Brid., *Grimmia plagiopodia* Hedw., *Orthogrimmia sessitana* (De Not.) Ochyra, *Hennediella heimii* (Hedw.) R.H. Zander and *Bryum pseudotriquetrum* (Hedw.) P. Gaert., that currently show a disjunct distribution in continental Antarctica as well as in the Antarctic Peninsula (Ochyra et al., 2008), may similarly have used dispersal routes that connected Victoria Land and the Antarctic Peninsula, such as through the now heavily glaciated coastal regions of the Amundsen and Bellingshausen Seas or along the Transantarctic Mountains. The history of *B. argenteum* populations identified in the current study is compatible with the availability of an intra‐Antarctic dispersal route as suggested by Hill, Bolton, and Haywood (2017). During the Pliocene warming event, the extent of ice‐free areas suitable for potential direct dispersal in Antarctica was very large, extending from the western side of the Antarctic Peninsula, through Ellsworth Land and Marie Byrd Land, up to the Edward VII Peninsula (i.e., across the Scotia and Byrd sectors), the Rockefeller Mountains, and Victoria Land. This scenario is compatible with the contemporary and disjunct geographic distribution of *B. argenteum* primarily in the AP and Victoria Land (Cannone et al., 2013; Ochyra et al., 2008). This species has also been reported from three widely separated coastal sites around continental Antarctica, in Marie Byrd Land, Dronning Maud Land, and Enderby Land (Ochyra et al., 2008). The former is most relevant to the hypothesis of an intra‐Antarctic dispersal route from Victoria Land to the AP, potentially either along the Transantarctic Mountains or the Marie Byrd Land and Ellsworth Land coastline.

### 4.3 Geographic distribution of Antarctic exclusive haplotypes

Antarctic exclusive haplotypes (Table 1 and Table S1) were present at four locations in Victoria Land (*Hap2* at Cape Hallett, *Hap5* at Gondwana, *Hap11* at Edmonson Point, and *Hap68* at Lamplugh Island), ranging from 72 to 75°S. Although there were a more limited number of samples available from the Antarctic Peninsula, a similar pattern was found, with six exclusive haplotypes detected at five locations (James Ross Island, Danco Coast, Léonie Island, Marguerite Bay, and Cockburn Island) between 63 and 67°S. This distribution of novel and exclusive haplotypes may indicate the existence of “hot‐spot” biodiversity areas around Antarctica.

Pisa et al. (2014) hypothesized that geothermal habitats might have played a role in the longer‐term regional persistence of *B. argenteum*, but indicated that more investigations were needed. Although, as noted above, geothermal areas associated with volcanic activity may have favored the persistence of ice‐free ground locally, providing refugia for some terrestrial biota in Antarctica (Convey & Smith, 2006; Fraser et al., 2014), our data indicate that the geothermal refugia hypothesis may not be the most appropriate hypothesis to apply to *B. argenteum*, as previously envisaged by Ochyra et al. (2008). Indeed, assuming niche conservatism (the tendency of species to retain their niches and related ecological traits over time; Wiens et al., 2010), the contemporary distribution of *B. argenteum* in Antarctica shows no close relationship to geothermal sites. In Victoria Land, the species is absent from Mount Melbourne fumaroles (also Mount Rittmann) (Bargagli, Broady, & Walton, 1996) and there is one specimen record from 1965 (Ochyra et al., 2008) and one more recent sequence record (98% similarity) in an eDNA study (Fraser, Connell, Lee, & Cary, 2018) for the Mt. Erebus area. The species is specifically recorded on Deception Island in the maritime Antarctic, although this latter location has a relatively recent age, originating only some tens of thousands of years ago (Smellie, 1988). Further, the in situ persistence of this species in geothermal refugia would lead to expectation of differentiation of endemic haplotypes with localized distribution associated with these geothermal areas. However, the haplotypes occurring relatively close to geothermal areas are characterized by the widespread *Hap1*, associated with *Hap2* in the Cape Hallett area and *Hap3* at Beaufort Island, both differentiated from *Hap1* by only a single mutational step. *Hap 1* also occurred in Deception Island. We therefore suggest that geothermal areas were not the primary means of in situ persistence of *B. argenteum* in Antarctica.

An alternative possibility supporting in situ persistence might be the availability of nunataks, such as those currently present in northern Victoria Land (e.g., the Deep Freeze Range, 74°S, 163°E), which have been continuously exposed and largely ice‐free since at least 5–7 Ma (Di Nicola et al., 2012). The potential role of such nunataks for the long‐term persistence of *B. argenteum* in North Victoria Land could be consistent with the higher haplotype diversity being found around 74°S in the latitudinal transect, including both ancient (*Hap1* and *Hap10*) and exclusive (*Hap11* and *Hap5*) haplotypes. Using cosmogenic dating, Oberholzer et al. (2003) demonstrated that Pleistocene glacial advances reached elevations up to 780 m, leaving several sites located above this threshold up to 1,050 m deglaciated since at least 5.3 Ma. These would provide potential refugia allowing survival during Pleistocene glaciations (see also Winkworth et al., 2015). Given niche conservatism, as the contemporary distribution of *B. argenteum* includes elevations up to 1,050 m in Victoria Land (Ochyra et al., 2008), the survival of this species in nunatak refugia in this region can be hypothesized.

Long‐term in situ persistence of *B. argenteum* in Antarctica could have been achieved both in refugia or, potentially, through cryptobiosis (long‐term survival in a biologically inactive state). An example of this has been demonstrated recently at Rothera Point (AP), where clump‐forming mosses survived using cryptobiosis over six centuries of cold‐based glacier burial and, after re‐exposure due to glacier retreat, were able to return to a metabolically active state (Cannone et al., 2017). A similar inference was made by La Farge, Williams, and England (2013) in the Canadian High Arctic, as well as by Roads, Longton, and Convey (2014) at Signy Island (South Orkney Islands) within bank‐forming mosses over a period of up to two millennia preserved in permafrost. In Victoria Land, cold‐based glaciers were widespread between c. 5–7 Ma and 2.5 Ma (Di Nicola et al., 2012; Smellie et al., 2014), providing the possibility of survival during glaciations through cryptobiosis, although it has to be accepted that the duration of glacial periods (tens to hundreds of thousands of years) is considerably longer than any currently available demonstration of cryptobiotic survival other than in microorganisms (Convey et al., 2020).

In conclusion, this study has provided evidence that *B. argenteum* occurred in Antarctica and Africa (as well as possibly Asia and North America) soon after the species’ origin and before the differentiation of its major constituent clades. Its earliest intra‐Antarctic dispersal and diversification occurred during Pliocene, likely favored by paleoclimatic warming during this era, with this radiation also leading to colonization of the sub‐Antarctic islands (around 46°S latitude) through migration from Antarctica. More recent dispersal and diversification within Antarctica also likely occurred during warm interglacial periods in the Pleistocene, a geological period that most likely shape its extant worldwide distribution. Contemporary diversity in Antarctic *B. argenteum* has been influenced both by multiple long‐distance dispersal events and by local persistence and in situ diversification, determining its population genetic structure within the region. Our analyses confirm that *B. argenteum* shows strong intra‐Antarctic connectivity (scenario I), rejecting the hypothesis that genetic structure within this species would match the distribution of Antarctic Conservation Biogeographic Regions (scenario II). The lack of genetic differentiation between AP and CA may be explained by the existence of intra‐Antarctic dispersal routes at earlier times.

## CONFLICT OF INTEREST

All authors declare that they do not have any conflict of interest.

## AUTHOR CONTRIBUTION

**Serena Zaccara:** Formal analysis (lead); Methodology (equal); Writing‐original draft (equal). **Jairo Patiño:** Formal analysis (equal); Software (equal); Writing‐review & editing (equal). **Peter Convey:** Writing‐review & editing (equal). **Isabella Vanetti:** Formal analysis (equal); Writing‐review & editing (equal). **Nicoletta Cannone:** Conceptualization (lead); Formal analysis (equal); Funding acquisition (lead); Writing‐original draft (lead); Writing‐review & editing (equal).

## Supporting information

Supplementary Material

## Data Availability

All molecular data are deposited in Genbank and the accession numbers of original sequences produced in this study are reported in Table 1.
