# Supplementary material for: Multiple colonization and dispersal events hide the early origin and induce a lack of genetic structure of the moss Bryum argenteum in Antarctica
Source: Ecol Evol. 2020 Aug 5;10(16):8959–75. doi: 10.1002/ece3.6601 (PMC7452785; doi:10.1002/ece3.6601)
Supplement: Supplementary file 1 — Supplementary Material [file ECE3-10-8959-s001.docx]

**Supplementary material**

**Table S1**. ITS nrDNA sequences available in GenBank, detailing geographic data (i.e. Continent, sample ID, collection localities, geographic coordinates) and molecular features (i.e. haplotype, bp length, clade and GenBank accession numbers). All sequences are refereed to specific with specific source references. Antarctic biogeographic regions area: MASSI = Maritime Antarctic South Sandwich Islands; NWAP = North-West Antarctic Peninsula; CSAP= Central-South Antarctic Peninsula; NVLCA = Northern Victoria Land Continental Antarctica; SVLCA = Southern Victoria Land Continental Antarctica.

| Continent | Sample ID | Localities | Latitude - Longitude | Hap  ITS | GB code | Ref. |
| --- | --- | --- | --- | --- | --- | --- |
| **Antarctica***  MASSI | AAS207A | Candlemas I | 57°07’ S 26°70’W | Hap14 | KJ409568 | Pisa et al. (2014) |
| MASSI | AAS202B | Candlemas I | 57°07’ S 26°70’W | Hap14 | KJ409563 | Pisa et al. (2014) |
| NWAP | AAS40 | Deception I | 62°18’ S 58°58’W | Hap1 | KJ409570 | Pisa et al. (2014) |
| NWAP | AAS1750 | King George I | 62°18’ S 58°58’W | Hap1 | KJ409559 | Pisa et al. (2014) |
|  |  |  |  |  |  |  |
| NEAP | AAS7922 | Cockburn Island | 64°20’S 56°85’ W | Hap8 | KJ409560 | Pisa et al. (2014) |
| NWAP | AAS4176 | Danco Coast | 64°68’S 62°63’ W | Hap26 | KJ409564 | Pisa et al. (2014) |
| NWAP | AAS8812 | Danco Coast | 64°68’S 62°63’ W | Hap26 | KJ409567 | Pisa et al. (2014) |
| CSAP | AAS4713 | Jenny Island | 67°43’S 63°38 W | Hap1 | KJ409561 | Pisa et al. (2014) |
|  |  |  |  |  |  |  |
| NVLCA | SH27 | Cape Hallett | 72°18’S 170°18’E | Hap2 | GU907068 | Hills et al. (2010) |
| NVLCA | SH21 | Cape Hallett | 72°18’S 170°18’E | Hap1 | GU907066 | Hills et al. (2010) |
| NVLCA | SH17 | Cape Hallett | 72°18’S 170°18’E | Hap1 | GU907067 | Hills et al. (2010) |
| NVLCA | EP | Edmonson Point | 74°20’S 164°30’E | Hap1 | AY611430 | Skotnicki et al. (2005) |
| NVLCA | AAS11794 | Victoria Land | 74°33’S 165°13’E | Hap10 | KJ409562 | Pisa et al. (2014) |
| NVLCA | SH47 | Marble Point | 76°43’S 163°83’E | Hap1 | GU907069 | Hills et al. (2010) |
| SVLCA | 20333 | Beaufort Island | 76°91’S 166°90’E | Hap1 | GU907063 | Hills et al. (2010) |
| SVLCA | BIE1 | Beaufort Island | 76°91’S 166°90’E | Hap1 | GU907064 | Hills et al. (2010) |
| SVLCA | 15f | Beaufort Island | 77°00’S 167°00’E | Hap3 | AY611431 | Skotnicki et al. (2005) |
| SVLCA | 20317 | Granite Harbour | 77°00’S 162°50’E | Hap1 | GU907065 | Hills et al. (2010) |
| SVLCA | D63 | Granite Harbour | 77°00’S 162°30’E | Hap1 | AY611434 | Skotnicki et al. (2005) |
| SVLCA | RH8/2 | Cape Bird | 77°21’S 166°43’E | Hap1 | GU907070 | Hills et al. (2010) |
| SVLCA | BB-1 | Botany Bay | 77°S 162°E | Hap1 | KT343959 | No pub |
| SVLCA | BM001102063 | Lake Vanda | 77°31’S 161°34’E | Hap64 | KX981163 | Rankin et al. (2017) |
| SVLCA | C474 | Cape Royds | 77°35’S 166°10’E | Hap1 | AY611433 | Skotnicki et al. (2005) |
| SVLCA | P171 | Canada Glacier | 77°58’S 163°25’E | Hap1 | AY611432 | Skotnicki et al. (2005) |
| SVLCA | CD6 | Cape Chocolate | 77°95’S 164°50’E | Hap1 | AY611429 | Skotnicki et al. (2005) |
| SVLCA | SH34 | Garwood Valley | 78°05’S 164°16’E | Hap1 | GU907072 | Hills et al. (2010) |
| SVLCA | MG20/10 | Miers valley | 78°08’S 164°75’E | Hap1 | GU907071 | Hills et al. (2010) |
| **Europe** | 40256B | Spain Granada | 36°80’N 3°21’W | Hap55 | KC493867 | Pisa et al. (2014) |
|  | 40274A | Spain Granada | 37°07’N 3°38’W | Hap67 | KC493852 | Pisa et al. (2014) |
|  | 40235R | Spain Granada | 37°09 N 3°40 W | Hap27 | KC493863 | Pisa et al. (2014) |
|  | 40221R | Spain Granada | 36°84 N 3°51W | Hap19 | KC493873 | Pisa et al. (2014) |
|  | MUB44625 | Norway Svalbard | 78°91’N 11°93’E | Hap1 | KF952782 | Pisa et al. (2014) |
|  | SB185204 | Denmark Faroe | 62°01’N 06°75’W | Hap19 | KF952834 | Pisa et al. (2014) |
|  | MUB 1097 | France | 43°81’N 18°43’E | Hap19 | KF952835 | Pisa et al. (2014) |
|  | MUB 44630 | Germany | 47°84’N 08°01’E | Hap19 | KF952832 | Pisa et al. (2014) |
|  | MUB 44637 | Germany | 47°86’N 08°02’E | Hap19 | KF952824 | Pisa et al. (2014) |
|  | MUB 44638 | Germany | 47°85’N 08°03’E | Hap19 | KF952825 | Pisa et al. (2014) |
|  | MUB 44639 | Germany | 47°85’N 08°03’E | Hap19 | KF952826 | Pisa et al. (2014) |
|  | MUB 44640 | Germany | 47°85’N 08°03’E | Hap19 | KF952827 | Pisa et al. (2014) |
|  | MUB 44649 | Germany | 49°46’N 08°98’E | Hap19 | KF952830 | Pisa et al. (2014) |
|  | MUB 44650 | Germany | 49°46’N 08°98’E | Hap19 | KF952837 | Pisa et al. (2014) |
|  | MUB 44651 | Germany | 49°24’N 08°65’E | Hap19 | KF952831 | Pisa et al. (2014) |
|  | MUB 44645 | Germany | 52°44’N 13°58’E | Hap19 | KF952828 | Pisa et al. (2014) |
|  | MUB 44647 | Germany | 52°51’N 13°39’E | Hap19 | KF952829 | Pisa et al. (2014) |
|  | MUB 12010 | Greece | 38°48’N 22°50’E | Hap19 | KF952836 | Pisa et al. (2014) |
|  | S B176550 | Norway | 70°78’N 23°33’E | Hap19 | KF952822 | Pisa et al. (2014) |
|  | 40238B | Spain | 36°84’N 06°50’W | Hap19 | KC493862 | Pisa et al. (2014) |
|  | MUB 44659 | Spain | 42°89’N 05°97’W | Hap19 | KF952823 | Pisa et al. (2014) |
|  | MUB 44644 | Germany | 52°50’N 13°33’E | Hap24 | KF952867 | Pisa et al. (2014) |
|  | MUB 12158 | Greece | 38°90’N 22°83’E | Hap29 | KF952794 | Pisa et al. (2014) |
|  | DT H03-186 | Ireland | 53°61’N 09°30’W | Hap29 | KF952788 | Pisa et al. (2014) |
|  | MUB 44652 | Spain | 40°42’N 05°29’W | Hap29 | KF952796 | Pisa et al. (2014) |
|  | MUB 44653 | Spain | 40°42’N 05°29’W | Hap29 | KF952789 | Pisa et al. (2014) |
|  | MUB 44652B | Spain | 40°42’N 05°29’W | Hap29 | KF952795 | Pisa et al. (2014) |
|  | MUB 44660 | Spain | 42°89’N 05°97’W | Hap29 | KF952790 | Pisa et al. (2014) |
|  | MUB 44663 | Spain | 08°02’N 01°16’W | Hap29 | KF952791 | Pisa et al. (2014) |
|  | MUB 44664 | Spain | 37°58’N 01°42’W | Hap29 | KF952792 | Pisa et al. (2014) |
|  | MUB 44641 | France | 47°90’N 07°10’E | Hap30 | KF952798 | Pisa et al. (2014) |
|  | DTH08-670 | UK Great Britain | 52°96’N 00°55’E | Hap31 | KF952799 | Pisa et al. (2014) |
|  | MUB 44668 | Spain | 40°59’N 03°98’W | Hap35 | KF952803 | Pisa et al. (2014) |
|  | MUB 44662 | Spain | 42°89’N 05°97’W | Hap39 | KF952838 | Pisa et al. (2014) |
|  | MUB 44665 | Spain | 39°89’N 04°10’E | Hap45 | KF952820 | Pisa et al. (2014) |
|  | MUB 44658 | Germany | 49°41’N 08°70’E | Hap45 | KF952841 | Pisa et al. (2014) |
|  | MUB 44648 | Germany | 49°41’N 08°70’E | Hap45 | KF952840 | Pisa et al. (2014) |
|  | MUB 44646 | Germany | 52°51’N 13°89’E | Hap45 | KF952852 | Pisa et al. (2014) |
|  | MUB 44661 | Spain | 42°89’N 05°97’W | Hap49 | KF952819 | Pisa et al. (2014) |
|  | S B178200 | Sweden | 59°78’N 14°36’E | Hap48 | KF952818 | Pisa et al. (2014) |
|  | MUB44666 | Spain | 40°83’N 03°83’W | Hap53 | KF952868 | Pisa et al. (2014) |
|  | MUB44667 | Spain | 40°83’N 03°83’W | Hap53 | KF952869 | Pisa et al. (2014) |
|  | DTH01-553 | France | 42°73’N 00°05’W | Hap57 | KF952881 | Pisa et al. (2014) |
|  | MUB 44642 | France | 47°90’N 07°10’E | Hap58 | KF952882 | Pisa et al. (2014) |
|  | MUB 44628 | Germany | 47°84’N 08°01’E | Hap65 | KF952847 | Pisa et al. (2014) |
|  | MUB 44643 | Portugal | 40°53’N 07°26’W | Hap65 | KF952848 | Pisa et al. (2014) |
|  | MUB 44657 | Portugal | 40°53’N 07°26’W | Hap65 | KF952846 | Pisa et al. (2014) |
|  | MUB 44626 | Germany | 47°83’N 08°01’E | Hap66 | KF952856 | Pisa et al. (2014) |
|  | MUB 44627 | Germany | 47°83’N 08°01’E | Hap66 | KF952857 | Pisa et al. (2014) |
|  | MUB 44629 | Germany | 47°84’N 08°01’E | Hap66 | KF952864 | Pisa et al. (2014) |
|  | MUB 44631 | Germany | 47°86’N 08°02’E | Hap66 | KF952862 | Pisa et al. (2014) |
|  | MUB 44632 | Germany | 47°86’N 08°02’E | Hap66 | KF952863 | Pisa et al. (2014) |
|  | MUB 44633 | Germany | 47°86’N 08°02’E | Hap66 | KF952858 | Pisa et al. (2014) |
|  | MUB 44634 | Germany | 47°86’N 08°02’E | Hap66 | KF952859 | Pisa et al. (2014) |
|  | MUB 44635 | Germany | 47°86’N 08°02’E | Hap66 | KF952860 | Pisa et al. (2014) |
|  | MUB 44636 | Germany | 47°86’N 08°02’E | Hap66 | KF952861 | Pisa et al. (2014) |
| **Asia** | E00416780 | Sri Lanka | 06°95’N 80°10’E | Hap1 | KF952781 | Pisa et al. (2014) |
|  | MSI06-1810 | Russia | 44°35’N 146°26’E | Hap19 | KF952833 | Pisa et al. (2014) |
|  | E00416777 | Taiwan | 23°81’N 121°23’E | Hap32 | KF952800 | Pisa et al. (2014) |
|  | CAS 995161 | Taiwan | 24°12’N 121°21’E | Hap33 | KF952801 | Pisa et al. (2014) |
|  | VEF08-162 | Russia | 71°88’N 110°78’E | Hap61 | KF952887 | Pisa et al. (2014) |
|  | NY 1229461 | Sumatra | 03°18’N 98°44’E | Hap37 | KF952805 | Pisa et al. (2014) |
|  | NY 1229462 | Thailand | 14°53’N 101°36’E | Hap41 | KF952810 | Pisa et al. (2014) |
|  | E 00477214 | China | 27°68’N 98°30’E | Hap45 | KF952815 | Pisa et al. (2014) |
|  | CAS 1140938 | China | 28°16’N 101°22’E | Hap45 | KF952849 | Pisa et al. (2014) |
|  | NY1229460 | Papua New Guinea | 05°75’S 145°03’E | Hap50 | KF952821 | Pisa et al. (2014) |
|  | MSI00-29 | Russia | 61°13’N 138°05’E | Hap51 | KF952855 | Pisa et al. (2014) |
|  | E00477222 | China | 37°11’E 102°31’E | Hap52 | KF952865 | Pisa et al. (2014) |
|  | E00477223 | China | 28°63’N 98°73’E | Hap52 | KF952866 | Pisa et al. (2014) |
|  | NY1229472 | China | 33°00’N 97°27’E | Hap54 | KF952873 | Pisa et al. (2014) |
|  | CAS1140922 | China | 30°17’N 100°00’E | Hap54 | KF952875 | Pisa et al. (2014) |
|  | GMUW94164-30 | China | 29°05’N 93°98’E | Hap54 | KF952874 | Pisa et al. (2014) |
|  | E 00477221 | China | 36°97’N 99°90’E | Hap56 | KF952885 | Pisa et al. (2014) |
|  | CAS 1141190 | China | 29°22’N 101°45’E | Hap59 | KF952883 | Pisa et al. (2014) |
|  | E 00477231 | Nepal | 27°43’N 87°46’E | Hap60 | KF952886 | Pisa et al. (2014) |
|  | MSI0/111 | Altai Republic | 50°50’N 89°16’E | Hap62 | KF952888 | Pisa et al. (2014) |
|  | VO99/95 | Russia | 43°45’N 41°68’E | Hap65 | KF952813 | Pisa et al. (2014) |
| **Africa** | MUB5343 | South Africa | 32°76’S 18°00’E | Hap20 | KF952879 | Pisa et al. (2014) |
|  | EGR55(4100) | South Africa | 27°76’S 30°78’E | Hap25 | KF952880 | Pisa et al. (2014) |
|  | MUB 13090 | Morocco | 31°21’N 07°86’W | Hap29 | KF952793 | Pisa et al. (2014) |
|  | MUB 44656 | Canary Island | 28°35’N 00°72’W | Hap29 | KF952797 | Pisa et al. (2014) |
|  | MJWU5011a | Uganda | 01°08’N 29°80’E | Hap34 | KF952802 | Pisa et al. (2014) |
|  | REM10946 | Comores | 11°61’N 43°33’E | Hap36 | KF952804 | Pisa et al. (2014) |
|  | J Spe | Kenya | 00°41’N 36°61’E | Hap38 | KF952806 | Pisa et al. (2014) |
|  | JE144 | Tanzania | 04°25’S 37°99’E | Hap45 | KF952850 | Pisa et al. (2014) |
|  | MUB 44654 | Canary Island | 28°10’N 17°23’W | Hap55 | KF952785 | Pisa et al. (2014) |
|  | MUB 44653 | Canary Island | 28°10’N 17°23’W | Hap55 | KF952785 | Pisa et al. (2014) |
|  | MUB 44655 | Canary Island | 28°14’N 16°65’W | Hap55 | KF952844 | Pisa et al. (2014) |
| **sub-Antarctic islands (SAI)** | AAS235 | Prince Edward I. | 46°87’S 37°85’E | Hap16 | KJ409566 | Pisa et al. (2014) |
|  | AAS296 | Prince Edward I. | 46°92’S 37°75’E | Hap21 | KJ409569 | Pisa et al. (2014) |
|  | SH48 | Crozet Islands | 46°45’S 52°00’E | Hap13 | GU907058 | Hills et al. (2010) |
|  | AAS1687 | Possession I. | 46°42’S 51°83’E | Hap17 | KJ409558 | Pisa et al. (2014) |
| **North America** |  |  |  |  |  |  |
|  | NY69323 | Greenland | 70° 48’N 53°06’W | Hap1 | KF952783 | Pisa et al. (2014) |
|  | D Les | USA Connecticut | 41°85’N 72°51’W | Hap39 | KF952839 | Pisa et al. (2014) |
|  | CAS 989901 | USA California | 35°88’N 118°34’W | Hap40 | KF952809 | Pisa et al. (2014) |
|  | CAS 989203 | USA California | 37°16’N 119°09’W | Hap47 | KF952817 | Pisa et al. (2014) |
|  | NY710573 | USA New Mexico | 32°31’N 106°75’W | Hap51 | KF952853 | Pisa et al. (2014) |
|  | NY53227 | USA Utah | 37°79’N 112°80’W | Hap51 | KF952854 | Pisa et al. (2014) |
|  | CAS 1083159 | USA California | 37°51’N 119°60’W | Hap55 | KF952787 | Pisa et al. (2014) |
|  | CAS 1039823 | USA California | 36°06’N 118°59’W | Hap55 | KF952786 | Pisa et al. (2014) |
|  | CAS 1083051 | USA California | 36°09’N 121°44’W | Hap55 | KF952845 | Pisa et al. (2014) |
|  | CAS 1047424 | USA California | 33°77’N 116°67’W | Hap55 | KF952843 | Pisa et al. (2014) |
|  | CAS 1047515 | USA California | 34°22’N 117°06’W | Hap55 | KF952842 | Pisa et al. (2014) |
|  | CAS 1113251 | USA California | 35°85’N 117°38’W | Hap55 | KF952870 | Pisa et al. (2014) |
|  | CAS 957141 | USA California | 35°56’N 118°95’W | Hap55 | KF952872 | Pisa et al. (2014) |
|  | CAS 993294 | USA California | 36°73’N 118°85’W | Hap55 | KF952871 | Pisa et al. (2014) |
|  | NY 321062 | USA Alaska | 57°15’N 170°25’W | Hap56 | KF952876 | Pisa et al. (2014) |
|  | NY 635904 | Dominican Rep | 18°30’N 71°701W | Hap45 | KF952851 | Pisa et al. (2014) |
|  | NY 635898 | Dominican Rep | 18°81’N 70°61’W | Hap43 | KF952812 | Pisa et al. (2014) |
| **South America** | AAS5437 | Falkland Islands | 51°70’S 57°85’W | Hap12 | KJ409565 | Pisa et al. (2014) |
|  | A28 | Chile | 54°95’S 57°63’W | Hap19 | KF952808 | Pisa et al. (2014) |
|  | NY1229478 | Bolivia | 17°65’S 64°80’W | Hap44 | KF952814 | Pisa et al. (2014) |
|  | NY1229481 | Ecuador | 00°38’N 78°31’W | Hap46 | KF952816 | Pisa et al. (2014) |
|  | NY1229483 | Colombia | 00°95’N 77°81’W | Hap56 | KF952884 | Pisa et al. (2014) |
|  | NY1229477 | Bolivia | 16°61’S 68°07’W | Hap39 | KF952807 | Pisa et al. (2014) |
|  | NY1229480 | Brazil | 13°25’S 41°90’W | Hap42 | KF952811 | Pisa et al. (2014) |
| **Oceania** | SH56 | Australia | 36°47’S 146°03’E | Hap15 | GU907057 | Hills et al. (2010) |
|  | SH68 | Australia | 36°52’S 147°15’E | Hap18 | GU907061 | Hills et al. (2010) |
|  | SH42 | Australia | 35°18’S 149°08’E | Hap23 | GU907059 | Hills et al. (2010) |
|  | SH43 | New Zealand | 43°05’S 172°07’E | Hap15 | GU907056 | Hills et al. (2010) |
|  | A099 | New Zealand | 37°34’S 175°09’E | Hap24 | GU907062 | Hills et al. (2010) |
|  | SH16 | New Zealand | 35°53’S 175°28’E | Hap22 | GU907060 | Hills et al. (2010) |

**Table S2.** Setting of Bayesian reconstructions in BEAST

|  | **Calibration I based on angiosperm´s substitution rates** | | | |  | **Calibration II based on moss´s substitution rates** | | | |
| --- | --- | --- | --- | --- | --- | --- | --- | --- | --- |
|  | Path Sampling (PS) | | Stepping-Stone (SS) | |  | Path Sampling (PS) | | Stepping-Stone (SS) | |
|  | ln (MLE) | 2ln (BF) | ln (MLE) | 2ln (BF) |  | ln (MLE) | 2ln (BF) | ln (MLE) | 2ln (BF) |
| STYL | -3857.135 | 85.500 | -3858.488 | 88.082 |  | -3098.363 | 769.396 | -3098.285 | 768.858 |
| STBD | -3825.843 | 22.916 | -3825.907 | 22.920 |  | -2988.322 | 549.314 | -2931.602 | 435.491 |
| STCS | -3816.575 | 4.381 | -3817.335 | 5.776 |  | -3061.397 | 695.463 | -3038.723 | 649.733 |
| STCBS | -3829.057 | 29.345 | -3829.891 | 30.888 |  | -2910.195 | 393.060 | -2837.529 | 247.345 |
| URCLYL | -3850.614 | 72.459 | -3852.416 | 75.937 |  | -2788.687 | 150.043 | -2788.651 | 149.590 |
| URCLBD | -3824.946 | 21.123 | -3825.311 | 21.729 |  | -2790.982 | 154.633 | -2791.056 | 154.398 |
| **URCLCS** | **-3814.385** | **0.000** | **-3814.447** | **0.000** |  | **-2713.665** | **0.000** | **-2713.856** | **0.000** |
| URCLCBS | -3816.096 | 3.423 | -3816.588 | 4.283 |  | -2714.388 | 1.445 | -2714.762 | 1.811 |

**Table S3.** Parameters of ancestral area estimations based on the two time-calibrated MCC trees obtained from BEAST (calibration I and II) performed using package BioGeoBEARS

| CALIBRATION I | LnL | numparams | d | e | AICc | AICc_wt |
| --- | --- | --- | --- | --- | --- | --- |
| DEC | -257 | 2 | 0.084 | 0.073 | 518.3 | 0.0003 |
| **DIVALIKE** | **-249.6** | **2** | **0.093** | **4.10 10^-9^** | **503.3** | **0.5** |
| BAYAREALIKE | -292.7 | 2 | 0.12 | 1.02 | 589.6 | 9.30 10^-20^ |
|  |  |  |  |  |  |  |
| CALIBRATION II | LnL | numparams | d | e | AICc | AICc_wt |
| DEC | -252 | 2 | 0.11 | 0.1 | 508.2 | 0.19 |
| **DIVALIKE** | **-251.5** | **2** | **0.084** | **0.046** | **507.2** | **0.31** |
| BAYAREALIKE | -290.4 | 2 | 0.13 | 1 | 585 | 4.00 10^-18^ |


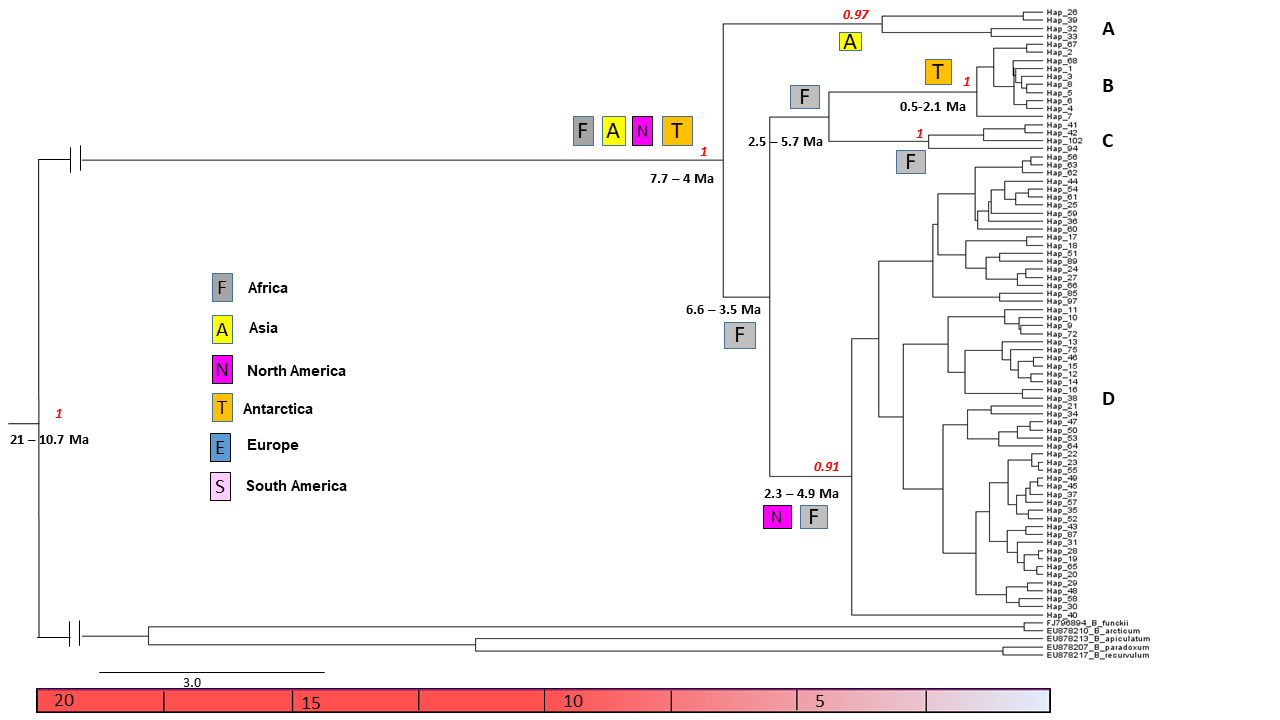


**Figure 1S**. BEAST chronogram of *B. argenteum* ITS nrDNA haplotypes based on angisperm’ substitution rate under the best fitting model (see table S2). The geographic distribution of haplotypes is detailed in Table 1 and Table S1 (supplementary material). Results of ancestral areas analysis based on DIVALIKE biogeographical model (see Table S3 supplementary material) are also reported. Significant posterior probability values at nodes (pp > 0.90) are indicated. The 95% highest posterior density (HPD) intervals are provided in Table 2. The geographic origins are Antarctica (T); Africa (F); Asia (A); North America (N); South America (S); Europe (E).
